# Direct-RT-qPCR Detection of SARS-CoV-2 without RNA Extraction as Part of a COVID-19 Testing Strategy: From Sample to Result in One Hour

**DOI:** 10.3390/diagnostics10080605

**Published:** 2020-08-18

**Authors:** Eva Kriegova, Regina Fillerova, Petr Kvapil

**Affiliations:** 1 Department of Immunology, OLGEN, Faculty of Medicine and Dentistry, Palacky University Olomouc and University Hospital, 77515 Olomouc, Czech Republic; regina.fillerova@fnol.cz; 2 Institute of Applied Biotechnologies a.s., 10800 Prague, Czech Republic; kvapil@ibiotech.cz

**Keywords:** 2019-nCoV, diagnostics, one-step direct RT-PCR, screening tests, point-of-care testing

## Abstract

Due to the lack of protective immunity in the general population and the absence of effective antivirals and vaccines, the Coronavirus disease 2019 (COVID-19) pandemic continues in some countries, with local epicentres emerging in others. Due to the great demand for effective COVID-19 testing programmes to control the spread of the disease, we have suggested such a testing programme that includes a rapid RT-qPCR approach without RNA extraction. The Direct-One-Step-RT-qPCR (DIOS-RT-qPCR) assay detects severe acute respiratory syndrome coronavirus 2 (SARS-CoV-2) in less than one hour while maintaining the high sensitivity and specificity required of diagnostic tools. This optimised protocol allows for the direct use of swab transfer media (14 μL) without the need for RNA extraction, achieving comparable sensitivity to the standard method that requires the time-consuming and costly step of RNA isolation. The limit of detection for DIOS-RT-qPCR was lower than seven copies/reaction, which translates to 550 virus copies/mL of swab. The speed, ease of use and low price of this assay make it suitable for high-throughput screening programmes. The use of fast enzymes allows RT-qPCR to be performed under standard laboratory conditions within one hour, making it a potential point-of-care solution on high-speed cycling instruments. This protocol also implements the heat inactivation of SARS-CoV-2 (75 °C for 10 min), which renders samples non-infectious, enabling testing in BSL-2 facilities. Moreover, we discuss the critical steps involved in developing tests for the rapid detection of COVID-19. Implementing rapid, easy, cost-effective methods can help control the worldwide spread of the COVID-19 infection.

## 1. Introduction

Coronavirus disease 2019 (COVID-19) is caused by the severe acute respiratory syndrome coronavirus 2 (SARS-CoV-2). COVID-19 was first identified in December 2019 in Wuhan, China. By the end of July 2020, more than 17 million people had been infected worldwide [1]. Due to the lack of protective immunity in the general population and the absence of effective antivirals and vaccines, the COVID-19 pandemic continues in some countries, with local epicentres emerging in others. Therefore, emphatic demand for effective COVID-19 testing programmes to control the spread of the disease has arisen. There are currently several approaches to COVID-19 diagnostics that are predominantly based on the detection of viral RNA isolated from nasopharyngeal swabs sampled in a viral transport medium by RT-qPCR [2]; on the other hand, serology or antibody tests are not suitable for diagnosing active COVID-19 infections [3].

With the increasing requirement for testing worldwide, inexpensive, fast and safe high-performance methods for detecting acute infection can help monitor outbreaks of COVID-19; optimise restrictions on public life, healthcare, travelling and business; and also reduce the isolation times of suspected patients. In addition, such high-throughput testing can be used for screening local epicentres and following up with individuals who have tested negative but are nevertheless suspected of having the disease.

We, therefore, have suggested an effective COVID-19 testing programme that includes a rapid RNA extraction-free RT-qPCR approach. For this purpose, we developed a Direct-One-Step-RT-qPCR (DIOS-RT-qPCR) assay, which possesses advantages in both speed and cost; it also solves the problem posed by a shortage of RNA isolation kits and addresses safety concerns. The newly developed DIOS-RT-qPCR assay is rapid, cost-effective and easy to perform under standard laboratory conditions and may also be utilised in large COVID-19 screening programmes and point-of-care solutions, not only to monitor outbreaks of infection.

## 2. Materials and Methods

A DIOS-RT-qPCR assay without RNA extraction was developed for the detection of SARS-CoV-2 RNA directly from a swab (nasopharyngeal, nasal) in a universal transport medium for viruses and bacteria (UTM, COPAN Diagnostics Inc., Murrieta, CA, USA). Our assay uses the novel SARS-CoV-2 sequences reported by the United States Centers for Disease Control, targeting two genetic sequences of the viral nucleocapsid (*N1*, *N2* genes) and the human RNase P (*RP* gene) as an internal control [4] (Table 1). The virus-specific probes were labelled by a dye–quencher pair FAM/BHQ1 probe and for human RNase P by Cy5/BHQ2 (TIB MOLBIOL GmbH, Berlin, Germany). Positive controls were prepared by spiking synthetic SARS-CoV-2 RNA controls (SARS-CoV-2 RNA control 1, Twist Bioscience, San Francisco, CA, USA) into standard human breast tumour total RNA (Takara Bio USA, Inc., Mountain View, CA, USA).

The DIOS-RT-qPCR assay was performed using an Xceed Fast One-step RT-qPCR Probe Kit (IABio, Prague, Czechia), which consisted of Xceed RTase 20× (an enzyme mixture of a thermostable and extremely active modified Moloney Murine Leukemia Virus (MMLV) reverse transcriptase, an advanced RNase inhibitor and advanced antibody-mediated hot start Taq DNA polymerase) and Xceed Fast One-step RT-qPCR Probe Mix (2×). The reaction was performed in a 30-μL reaction volume containing 16 μL of Master Mix and 14 μL of the analysed swab. The final concentrations in the reaction were as follows: 0.9× for the Xceed Fast One-step RT-qPCR Probe Mix and 1× for the Xceed RTase; for the concentrations of the forward and reverse primers and probes for the *N1*, *N2* and *RP* genes, see Table 1. In each run, negative (nuclease-free water) and positive (125 copies of synthetic SARS-CoV-2 RNA in an RNA carrier) controls were analysed. The thermal cycling conditions were as follows: 10 min at 45 °C for lysis and reverse transcription, 2 min at 95 °C for polymerase activation and 45 cycles of 5 s at 95 °C, 15 s at 55 °C (data acquisition) and 15 s at 72 °C. The analysis was performed using a real-time qTOWER3 system (Analytik Jena, Jena, Germany), a LightCycler 480 real-time PCR system (Roche, Basel, Switzerland) and a Rotor-Gene Q (QIAGEN, Hilden, Germany). A cycle threshold (Ct) value of <40 in the FAM channel (*N1/N2* genes) was indicative of a SARS-CoV-2-positive sample. Patient samples were expected to exhibit fluorescence amplification curves (Ct value < 40) in the Cy5 channel (human *RP* gene) to exclude those with insufficient human cellular material or loss of specimen integrity.

We also tested another kit with a reportedly high tolerance to swabs: the Luna Universal Probe One-Step RT-qPCR Kit (New England Biolabs, Inc., Ipswich, MA, USA), which consists of a Luna Universal One-Step Reaction Mix 2× and 20× Luna WarmStart RT Enzyme Mix (an enzyme mix of in-silico-designed modified reverse transcriptase and hot start Taq DNA polymerase). The best performance was obtained in the same set-up of a 30-μL reaction volume containing 16 μL of Master Mix and 14 μL of the analysed swab. The final concentrations in the reaction were as follows: 0.9× for the Luna Universal One-Step Reaction Mix and 1× for the Luna WarmStart RT Enzyme Mix; for the concentrations of the forward and reverse primers and probes for the *N1*, *N2* and *RP* genes, see Table 1. The thermal cycling conditions were as follows: 15 min at 55 °C for lysis and reverse transcription, 2 min at 95 °C for polymerase activation and 45 cycles of 5 s at 95 °C, 45 s at 55 °C (data acquisition) and 15 s at 72 °C.

The SARS-CoV-2 calibration curve was performed with a known amount of copies of synthetic SARS-CoV-2 RNA standard in UMT in the range of 2–750 copies/reaction volume and analysed via a LightCycler 480 real-time PCR system and a Rotor-Gene Q. The limit of detection (LOD) was estimated using real swab samples (n = 4), where the quantification of the number of copies was performed using a calibration curve with a known amount of copies of synthetic SARS-CoV-2 RNA standard in UMT. All measurements were performed on a LightCycler 480 real-time PCR system. After the amount of copies/mL in the real samples was calculated, all the samples were diluted to 53.57 × 10^3^ copies/mL in UMT, which translates to 750 copies/reaction volume. Then, a twofold dilution series of real swab samples was prepared, covering 2–750 copies/reaction volume. The analysis of a twofold dilution series of real swab samples was performed in duplicate using a real-time qTOWER3 system, a LightCycler 480 real-time PCR system and a Rotor-Gene Q.

In a set of samples (n = 40), viral RNA was isolated from swab samples using a viral RNA isolation kit from cell-free fluids (NucleoSpin RNA Virus, Macherey-Nagel, Düren, Germany). An In Vitro Diagnostic (IVD) CE-certified UltraGene Combo2Screen SARS-CoV-2 Assay Kit (ABL SA Group, Luxembourg, Luxembourg) was used to validate the DIOS-RT-qPCR results.

## 3. Results

First, the sensitivity of the DIOS-RT-qPCR assay was tested on a synthetic SARS-CoV-2 RNA control template diluted with UMT, reaching an LOD of two copies/reaction. The positive real swab samples (n = 4) were titered based on the Ct values using a standard curve prepared with SARS-CoV-2 synthetic RNA at the same concentration of 53.57 × 10^3^ copies/mL in UMT. Using a twofold dilution series of real swab samples (n = 4) diluted with UTM, the LOD was estimated at seven RNA copies/reaction based on the Ct value, a standard deviation of replicates and the shape of the fluorescence amplification curves in samples with a low viral load on a qTOWER3, a LightCycler 480 and a Rotor-Gene Q (Table 2). This translated to an LOD of lower than 550 virus copies/mL of swab medium. The assay’s reliability and accuracy were verified on five repeated positive samples, with a variability in Ct of <2%.

Regarding DIOS-RT-qPCR assay using both tested RT-qPCR kits (the Xceed Fast One-step RT-qPCR Probe Kit and the Luna Universal Probe One-Step RT-qPCR Kit), similar results were obtained with a variability in Ct of <3%.

**Figure 1:**
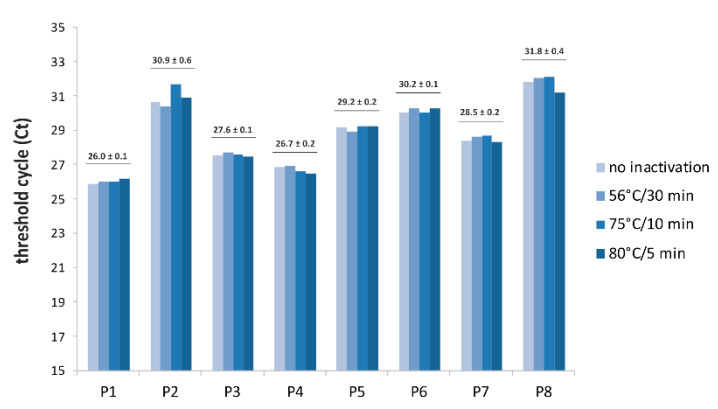
Influence of heat inactivation of severe acute respiratory syndrome coronavirus 2 (SARS-CoV-2) in swabs on DIOS-RT-qPCR results. Heat-inactivated and non-inactivated swabs were used to set up the RT-qPCR. For each patient sample (P), the mean and standard deviations of cycle threshold (Ct) using heat inactivation are given.

Second, we investigated the influence of swab pre-treatment by heating leading to the inactivation of SARS-CoV-2. The swab samples were incubated at 56 °C for 30 min, 75 °C for 10 min and 80 °C for 5 min; they were then used to set up the RT-qPCR reaction. Our RT-qPCR analysis revealed that most of the paired, inactivated and non-inactivated samples had the same Ct, irrespective of the inactivation profile used. Only about 5% of the inactivated samples resulted in higher Ct values, and 5% resulted in lower values (see Figure 1).

**Figure 2:**
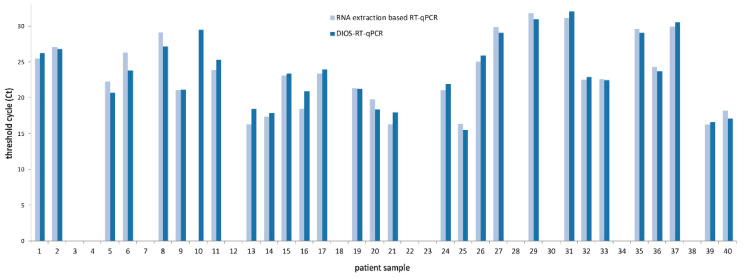
Results of the paired analysis of swab samples by DIOS-RT-qPCR (directly from swab) and an IVD CE-certified RT-qPCR kit (using extracted RNA).

Except for one sample returning positive from the swab and negative from the isolated RNA, a 98% concordance of results was observed between the DIOS-RT-qPCR and the IVD-validated kit in terms of Ct values and final positivity/negativity (Figure 2). The discordant sample, in which the viral RNA was lost during the RNA isolation step, was a weak positive with a high Ct. However, when RNA isolation was repeated using the RNA isolation kit from the other vendor, the result confirmed the sample’s SARS-CoV-2 positivity.

Moreover, our assay accurately analysed all samples from an external quality control run, the Coronavirus Outbreak EQA Pilot Study Challenge S2, which was provided by Quality Control for Molecular Diagnostics (QCMD) in April 2020 (results evaluation June 2020). This control run included eight samples in UTM, of which five were positive for SARS-CoV-2 and one was negative and two were positive for other coronaviruses (coronavirus OC43 and coronavirus NL63). The results obtained via the DIOS-RT-qPCR, the recently published direct RT-qPCR approach and the commercial QIAstat-Dx 2019-nCoV Respiratory Panel Assay (QIAGEN) [5] are shown in Table 3.

## 4. Discussion

Effective COVID-19 testing programmes must be designed as soon as possible to control the worldwide spread of COVID-19. To this end, rapid, cost-effective diagnostic tests need to be developed that simplify lengthy analytic steps and deliver results in a short time with the desired levels of sensitivity and specificity. Therefore, we developed and validated an RNA-isolation-free RT-qPCR assay that is fast, safe, cost-effective and achieves comparable sensitivity to the standard methods, delivering results within an hour of sample collection.

In the current state of SARS-CoV-2 diagnostics, the most limiting, time-consuming step is the isolation of viral RNA from swabs sampled in a viral transport medium prior to RT-qPCR. Although the unavailability of extraction kits and reagents has become less severe over time, this step is labour- and time-intensive and is associated with the risk of contamination, sample exchange and/or low virus recovery for some samples. The last factor is particularly relevant to samples with low viral load, as shown in our study. Therefore, the omission of the RNA extraction step may be vital to the development of rapid assays. In the absence of an RNA isolation kit, it is crucial to use enzymes that are tolerable of high levels of inhibitors [6]. In our DIOS-RT-qPCR assay, we used enzymes that tolerated a large volume of the swab (14 μL, 47% *v*/*v*), reaching comparable sensitivity to methods based on RNA extraction, which usually work with 3–5 μL of RNA isolates. The recent studies reporting on adding swab samples directly to the RT-qPCR reaction used small volumes of 3-μL [7] or 5-μL swabs [8,9,10], or 8 μL of fourfold diluted swab [5] in the RT-qPCR reaction, which resulted in limited sensitivity in samples with low viral loads. Using large volumes of the swab in RT-qPCR makes it is possible to detect patients in the acute phase of COVID-19 directly from the swab, where a median viral load of 1.69 × 10^5^ copies/mL (range 651 to 1.34 × 10^11^ copies/mL) in a nasopharyngeal swab is expected [11,12,13]. Moreover, performing the swab using a lower amount of viral transport medium may further enhance the sensitivity of our assay. Importantly, our reported DIOS-RT-qPCR assay fulfils the current FDA recommendation of 20 copies/reaction [14].

Another important activity related to the process of analysing infection specimens is protecting laboratory personnel. Large volumes of infectious material or high concentrations of live SARS-CoV-2 should be handled at a containment laboratory with inward directional airflow (BSL-3) [15]. Therefore, it would be more advantageous to work with inactivated SARS-CoV-2 samples, which would allow the clinical laboratories to handle patient samples in a BSL-2 environment instead of a BSL-3 one. Therefore, we tested whether the results of our assay would be influenced by the heat inactivation of SARS-CoV-2, which renders samples non-infectious, at 56 °C for 30 min, 75 °C for 10 min or 85 °C for 5 min before setting up the RT-qPCR reaction [16]. Like others [5,7,17], we observed a minimal impact on the RT-qPCR’s sensitivity in most of the paired inactivated and non-inactivated samples. Therefore, our DIOS-RT-qPCR assay may be used for swab specimens either immediately after collection or after heat inactivation of the virus, under the reported temperature profile, which would allow the assay set-up to be performed under BSL-2 conditions.

**Figure 3:**
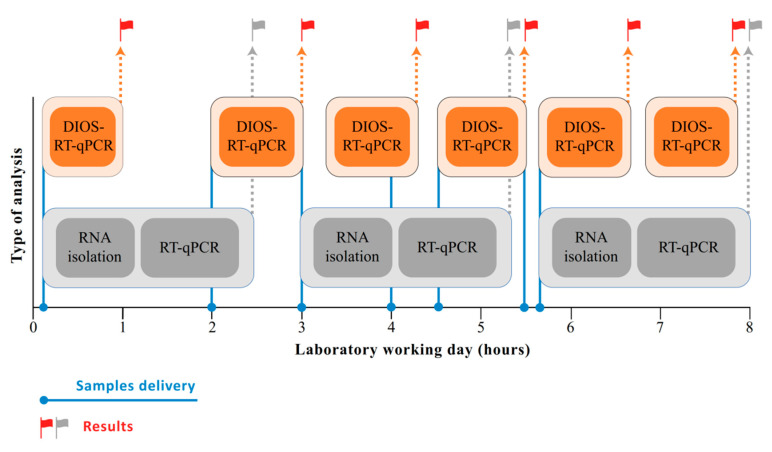
Timeline for laboratory analysis of delivered samples using RT-qPCR with/without RNA isolation.

Another important quality of effective testing is speed. Omitting RNA isolation will significantly shorten the time from sample to results (Figure 3). Another factor that influences test speed is which enzymes are used; rapid PCR polymerases may speed up reactions. Our test utilises fast enzymes capable of performing rapid RT-qPCR under standard laboratory conditions within 60 min on a fast real-time cycler qTOWER3 and 90 min on both a LightCycler 480 real-time PCR system and a Rotor-Gene Q. Moreover, when implementing our assay with fast enzymes as a point-of-care solution on a fast-cycling, real-time PCR platform, results may be obtained even faster.

Because, in most cases, the delivery of results from laboratories currently takes one to two days from sample delivery, there is an emerging need to develop fast assays. Utilising fast and robust enzymes and fast-cycling platforms, omitting RNA extraction steps, implementing automated data evaluation algorithms and/or novel alternative approaches to RT-qPCR may shorten result delivery to a few hours from sample delivery (Figure 3). Several alternative approaches to RT-qPCR have already been reported for the fast detection SARS-CoV-2, mainly based on Loop-mediated isothermal AMPlification (LAMP) [18,19,20]. The main advantage of the LAMP assay is its speed and minimal equipment requirements; however, it could still benefit from improvements vis-à-vis its sensitivity and specificity. Even more promising for COVID-19 diagnostics is a combination of the Specific High Sensitivity Enzymatic Reporter UnLOCKing (SHERLOCK), called SHERLOCK Testing in One Pot (STOP), which is a specific technique based on combining LAMP and CRISPR-mediated sequence-specific detection of the viral sequence [19]. Its reported LOD is 100 molecules of synthetic SARS-CoV-2 genomes in saliva or nasopharyngeal swab/reaction. A point-of-care assay version of SHERLOCK works with a simple lysis buffer for an extraction kit and a lateral flow dipstick, which delivers results from swabs or saliva in less than 40 min [20]. Other combinations of SHERLOCK, such as SHERLOCK and HUDSON Integration to Navigate Epidemics (SHINE), have been introduced to detect SARS-CoV-2 RNA from unextracted samples within 50 min, with a sensitivity of 10 copies/μL [21]. The first results show SHERLOCK’s great potential combinations for future COVID-19 screening and point-of-care-testing.

Nevertheless, there are several preanalytical steps that are vital to preventing false negatives in the diagnosis of SARS-CoV-2. Some authors report 30% false negatives in laboratory diagnostics of COVID-19, mainly due to various preanalytical steps [22]. Among the most important factors for avoiding false negatives are correct sampling and temperature during transport and all preanalytical steps. Nylon and dacron are suitable sampling materials, as they do not inhibit the PCR reaction [23]. The most suitable samples for the diagnosis of COVID-19 include nasopharyngeal and nasal swabs [22], saliva [24], sputum and lavage [25], in which a high viral load is detected in patients in the acute phase. Moreover, the time of sample-taking, sampling procedures and disease progression may affect RT-qPCR results [22]. In terms of temperature, SARS-CoV-2 remains stable at 4 °C with only around a 0.7 log-unit reduction of infection titre on day 14 [26]. However, the virus is sensitive to higher temperatures, which leads to degradation of its RNA [27]. Therefore, the recommended transport or handling of samples should be at 2–8 °C for up to 72 h; if a delay is expected, samples should be frozen at −70 °C [23].

**Figure 4:**
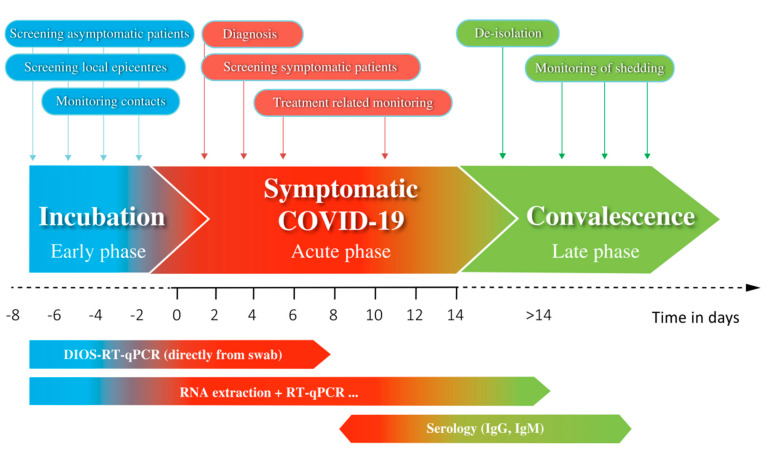
Design of a cost-effective Coronavirus Disease 2019 (COVID-19) testing programme. RNA-extraction-free assays (such as DIOS-RT-qPCR or others) should be implemented for screening acute infections and point-of-care solutions.

Taken together, the development of rapid RT-qPCR approaches that exclude RNA extraction or otherwise increase speed, is of great importance to controlling the spread of infection in the near future and should be part of an effective COVID-19 testing programme (Figure 4). Combinations of fast enzymes that have a high tolerance to inhibitors, as used in our DIOS-RT-qPCR, with fast-cycling instruments have great potential for implementation in effective COVID-19 testing programmes in the near future, not only as screening programmes (Figure 4) but also as point-of-care solutions (Table 4).

## 5. Conclusions

We have suggested an effective COVID-19 testing programme for the near future that includes a rapid RT-qPCR approach without RNA extraction. Compared to RT-qPCR using RNA isolation, DIOS-RT-qPCR detects SARS-CoV-2 directly from swabs, thus significantly reducing the sample-to-result time to less than one hour, simplifying the analytical process and reducing the risk of exposure to infectious samples while maintaining the high levels of sensitivity and specificity required of diagnostic tools. Our robust test is easy to perform under standard laboratory conditions in high-performance formats, which allows for a significant increase in test numbers. Our study further highlights the fast, easy, cost-effective and sensitive features of the COVID-19 testing programme necessary for the near future that may accelerate clinical decision-making.

## Figures and Tables

**Table 1:** Primer and probe sequences [4] and concentrations used for Direct-One-Step-RT-qPCR (DIOS-RT-qPCR) assay.

Name	Description	Oligonucleotide Sequence (5′ → 3′)	Final Concentration in Reaction
**2019-nCoV_N1**	Forward primer	GACCCCAAAATCAGCGAAAT	333 nM
	Reverse primer	TCTGGTTACTGCCAGTTGAATCTG	333 nM
	Probe	FAM-ACCCCGCATTACGTTTGGTGGACC-BHQ-1	83 nM
**2019-nCoV_N2**	Forward primer	TTACAAACATTGGCCGCAAA	200 nM
	Reverse primer	GCGCGACATTCCGAAGAA	200 nM
	Probe	FAM-ACAATTTGCCCCCAGCGCTTCAG-BHQ-1	50 nM
**RP**	Forward primer	AGATTTGGACCTGCGAGCG	133 nM
	Reverse primer	GAGCGGCTGTCTCCACAAGT	133 nM
	Probe	Cy5-TTCTGACCTGAAGGCTCTGCGCG-BHQ-2	33 nM

**Table 2:** Sensitivity of the DIOS-RT-qPCR assay in nasopharyngeal swabs. Patient swab samples (n = 4), titered to the same virus copies/volume, were analysed in a twofold dilution series using standard instruments. The mean and standard deviations of the Ct were calculated from data obtained in all analysed samples at given virus copies/reaction; samples were analysed in duplicate. ND: not detected.

Mean Ct ± SD			Virus Copies/ Reaction	Virus Copies/mL in Swabs
qTOWER3	LightCycler 480	Rotor-Gene Q		
25.9 ± 0.1	28.2 ± 0.2	24.0 ± 0.1	125	8.9 × 10^3^
26.8 ± 0.1	29.4 ± 0.2	25.2 ± 0.1	63	4.5 × 10^3^
27.5 ± 0.1	30.7 ± 0.1	25.7 ± 0.1	31	2.2 × 10^3^
28.3 ± 0.1	31.9 ± 0.2	26.9 ± 0.2	15.6	1.1 × 10^3^
29.1 ± 0.1	32.8 ± 0.1	27.9 ± 0.2	7.8	554
30.0 ± 0.2	33.6 ± 0.2	28.8 ± 0.2	3.9	277
30.6 ± 0.3	34.2 ± 0.3	29.4 ± 0.3	2	141
ND	ND	ND	0	0

**Table 3:** Results of Quality Control for Molecular Diagnostics (QCMD) EQA specimens obtained by DIOS-RT-qPCR and two RT-qPCR approaches [5]. For more details of the direct approach (using diluted heat lysed swab and TaqPath 1-Step RT-qPCR Master Kit, Thermo Fisher Scientific, Waltham, MA, USA) without RNA isolation and a QIAstat-Dx Respiratory 2019-nCoV Panel using extracted RNA and multiplex RT-PCR (QIAGEN), see [5]. ND: not detected.

QCMD EQA Specimens		DIOS-RT-qPCR Result		Study by Hasan et al. 2020 [5]	
*Sample ID*	*SARS-CoV-2 Result*	*SARS-CoV-2 Result*	*qTOWER3 (Ct)*	*QIAstat-Dx (Ct)*	*Direct Approach* *(Ct)*
S 01	Positive	Positive	24.4	34.0	35.5
S 02	Negative	Negative	ND	ND	ND
S 03	Positive	Positive	27.1	35.4	37.1
S 04	Negative	Negative	ND	ND	ND
S 05	Negative	Negative	ND	ND	ND
S 06	Positive	Positive	24.1	36.7	35.1
S 07	Positive	Positive	20.8	31.5	31.7
S 08	Borderline	Positive	30.2	ND	ND

**Table 4:** The parts of an effective COVID-19 testing programme: screening, point-of-care testing and diagnostics.

	Target Subjects	Result Expected	Key Requirements for Methods Used	DIOS-RT-qPCR
**Screening**	Local epicentres, contacts of positive cases, business, travelling, risk groups	Positive/negative for COVID-19	Fast, cost-effective, high throughput, easy performance	Yes, suitable
**Point-of-care testing**	Patients before emergent hospitalisation, surgery, doctor/dentist visits	Positive/negative for COVID-19	Ultra-fast, on-site	Yes, suitable
**Diagnostics**	Patients with suspected COVID-19	Viral load, quantification of pathogens, positive/negative for COVID-19 and other respiratory pathogens (panels of targets)	Analysis of multiple targets and genes, ultra-sensitive, quantitative and qualitative evaluation	Not intended
